# Social Differences in Health Behaviours among Jordanian Adolescents

**DOI:** 10.3390/ejihpe12080083

**Published:** 2022-08-19

**Authors:** Abdullah S. Alshammari, Bettina F. Piko, Tamás Berki, Kevin M. Fitzpatrick

**Affiliations:** 1 Doctoral School of Education, University of Szeged, 6725 Szeged, Hungary; 2 Department of Behavioral Sciences, University of Szeged, 6725 Szeged, Hungary; 3 Institute of Physical Education and Sport Sciences, University of Szeged, 6725 Szeged, Hungary; 4 Department of Sociology & Criminology, University of Arkansas, Fayetteville, AR 72701, USA

**Keywords:** health behaviours, social differences, adolescents

## Abstract

Social differences are evident in both developed and developing countries. During adolescence, there are limited differences in morbidity and mortality, but differences do appear in terms of health behaviours. This study aims to examine the relationship(s) between social differences and students’ health behaviours. A cross-sectional study was conducted in 2020 with a sample of high school students (N = 2741, aged 13–18 years) in Jordan. Besides descriptive statistics, bivariate logistic regression analysis was used to detect the odds risk for each social difference indicator. Females were engaged in more healthy dietary and hygienic behaviours and less engaged in smoking. Males were more physically active. Adolescents with a higher parental education level were more engaged in healthy behaviours; however, they drank carbonated soft drinks and ate fast food more often. Higher SES (socioeconomic status) self-evaluation was positively associated with eating breakfast and fruit and vegetables, being physically active, drinking carbonated soft drinks, eating fast food, and smoking. Our findings suggest that socioeconomic differences are important to understanding Jordanian adolescents’ health behaviours. While females tend to engage in more healthy behaviours, the role of parental education and perceived family affluence is not always beneficial in terms of adolescents’ dietary habits, hygienic behaviour, or smoking.

## 1. Introduction

Social differences and inequalities are important global public health issues [1]. However, social differences are not uniform across varying age groups; during adolescence, there are fewer differences in rates of morbidity and mortality, but differences do appear in terms of health behaviours [2,3]. Despite a growing number of studies internationally, a limited number of studies have been carried out on adolescents living in developing countries such as Jordan.

Social differences are usually found in terms of risky health behaviours, e.g., in relation to gender, parental status, and socioeconomic status (SES) self-assessment [4,5], whereas information about parents’ occupation or family income often have less predictive validity. Besides substance use, unhealthy dietary habits, lack of physical activity, and hygienic behaviour are the most common forms of risky behaviours that are in need of further exploration and intervention [6].

In terms of diet, a recent Iranian study found a relationship between parents’ education level and adolescents’ breakfast consumption [7]. While girls tend to eat more fruits and vegetables, they also skip breakfast more often, and boys typically consume more fast food and sugar-sweetened drinks [8]. In Germany, higher parental education has been shown to be associated with more fruit and vegetable intake [9]. Lower family SES has been associated with poorer diet quality [10]. An Italian study found that adolescents whose parents had a lower educational level consumed carbonated soft drinks and fast food less often [11]. Family affluence is also a positive predictor of these unhealthy dietary practices in different parts of the world [12,13].

Unlike nutrition, weekly physical activity among boys is greater than among girls internationally [8,14,15,16]. Physical activity is also more frequent among persons in higher social classes [16,17,18]. 

Besides these relationships, oral hygiene and hand washing are relevant health behaviours, particularly in developing countries. Gender [19,20], parental education [20,21], and family SES [21,22] can also contribute to social differences and inequalities. Children from lower-income and lower-educated households usually have poorer oral hygiene [23]. 

Finally, smoking is also a relevant health behaviour in developing countries [24]. Boys are usually more inclined to smoke than adolescent girls [25]. In addition, smoking is positively associated with socioeconomic adversity [26]. European data show higher adolescent smoking rates among low SES adolescents [27]. Adolescents from Saudi Arabia with a highly educated father and mother were less likely to use tobacco than their counterparts [28]. However, in a Korean study higher family SES was associated with smoking [18].

Despite a growing number of studies in this area, only a few have examined social differences in health behaviours among Jordanian adolescents. Adolescents at school report insufficient physical activity and low consumption of fruit or vegetables, as well as high consumption of fast foods, carbonated soft drinks, and sweets and inadequate tooth brushing [19]. Among the few Jordanian studies that have been conducted, Obeisat and Gharaibeh [29] reported that boys and adolescents with a higher family income were more likely to engage in physical activity [29]. Alshammari and Piko [30] found that fruit and vegetable consumption was associated with higher parental education, while fast food consumption was associated with higher family affluence. In terms of smoking, initiators of smoking were more likely to be males and those with less educated parents [31]. Hand washing was highest among Jordanian adolescent girls and those with highly educated mothers [21].

In terms of the Jordanian context, the culture has been shaped by Islamic religious and social norms, which encourage people to promote their health through preventive actions and healthy behaviours. Religious beliefs and practices and cultural norms often influence perceived behavioural control, social influence, and discussions about unhealthy behaviours such as smoking [32]. However, Jordan is facing rapid progress and is growing closer to Western countries, and socioeconomic changes could in turn affect the culture and norms, e.g., eating behaviour norms can be changed by the spread of fast food restaurants in cities [8]. Additionally, Jordanian adolescents tend to exhibit patterns of risky health behaviours such as physical inactivity or smoking, while gender can also make a difference in these fields more than in other places [33].

In comparison with other Arab countries, adolescent behaviour in this part of the world may reflect both traditional and global values depending on differences in historical, cultural, and socioeconomic conditions. For example, the frequency of smoking among 13–15 year olds is 15% or higher among boys in Bahrain, Kuwait, Syria, and Tunisia, and the rate is 10–15% among boys in Iraq, Lebanon, Qatar, Saudi Arabia, Sudan, and the UAE [34]. On the other hand, sedentary lifestyles are more common in countries such as the UAE, Jordan, and Oman when compared to Egypt, Libya, and Morocco [34]. The proportion at high risk of disordered eating ranged from 6.1% to 73.3% in a review, with particularly low levels in Israel and Egypt and the highest levels being in Oman, the UAE, and Kuwait [8,35,36].

For Jordan, there is a need to study school health services provided to students, which is currently suffering from shortages and stagnation in health services due to difficult economic, political, and social conditions. We need to detect social differences as risk factors in school health education in order to acquire knowledge regarding healthy behaviours, personal hygiene, balanced nutrition rules, and attention. Therefore, this study aims to examine the role of a set of social differences (namely, gender, parental education, and family affluence) in students’ health behaviours (such as dietary habits, hygienic behaviour, physical activity, and smoking) among Jordanian adolescents. We chose these measures since (a) these are the most relevant risk factors for adolescents’ future health [8,19,21,29,30,31] and (b) these measures are often used in other studies from the Arab world, and thus findings would be useful for comparison purposes and the determination of future directions in health education programmes. We primarily aimed to calculate odds ratios (OR) to determine the odds of these acting as risk factors, which is very practical in public health.

## 2. Materials and Methods

### 2.1. Participants and Procedure

Data were collected from public and private schools (from grades 8–12) in the Irbid governorate, which is located in northern Jordan and affiliated with the Jordanian Ministry of Education, using multistage cluster sampling in 2020. We selected Irbid city because it is the second largest city in Jordan (Department of Statistics [Jordan], 2022), all schools have computer laboratories, and they have good access to the internet to collect data. The Jordanian community has very small social, cultural, and economic differences among the population [37]. Thus, we think that this governorate is representative of other governorates in the country. A self-administered online questionnaire was given to 2741 students (13–18 years old). A total of 2760 students were asked to participate in our study, and 2741 students participated, giving a response rate of 99.1% (see Table 1 for details). The multi-stage random sampling technique was used to recruit the participants in this study. First districts and then schools were randomly selected, and finally we used class as the primary sampling unit (see details in Appendix A).

When obtaining human subjects approval from the Institutional Review Board at the University of Szeged, Hungary and the Ministry of Education in Jordan, we provided a simple explanation of the importance of the research while explaining that students had the freedom to participate in the research without any pressure from the school or parents and had the right to refuse to answer any question. Confidentiality and anonymity were ensured. All students recruited for the study were invited to voluntarily assent and obtain signed consent forms from their parents. Data were collected in the computer labs during the leisure or sports classes for the students through an online survey developed by the researcher using Google drive forms.

### 2.2. Measurements

The social variables included gender, SES self-evaluation, and father and mother’s education. Self-assessment by students identifying as lower-middle class or less, middle class, or upper-middle class or higher was used to assess family affluence. Parental education was assessed using categories of primary school or less, secondary school, or graduate/postgraduate for both the father and mother’s education. Since the number of working fathers is still much higher, we focused on the most appropriate variables for our research, such as paternal education, maternal education, and family affluence as SES measurements rather than job characteristics as the latter variables are often biased in studies.

The modified Arabic version of the Global School-based Student Health Survey (GSHS) questionnaire was used to measure a range of health behaviours [38]. The Arabic version of the GSHS has been demonstrated to have good validity and reliability across many different samples of Arabic countries including Jordan [39]. In this paper, we selected 10 variables to evaluate adolescent health behaviour, which are the most relevant behavioural risk factors in public health: dietary behaviour (6 items: not having enough food; eating breakfast every day; eating fruit daily; eating vegetables daily; drinking carbonated soft drink daily; eating fast food at a restaurant weekly); hygienic behaviour (2 items: cleaning/brushing teeth daily; hand washing before eating daily); tobacco use (1 item: smoking cigarettes monthly); and physical activity (1 item: physical activity weekly). We did not measure drinking since this is a very sensitive issue due to the prohibition of alcohol use in Muslim culture. All questions were phrased in the following way: ‘During the past 30 days, how often did you eat breakfast?’, etc. Responses were dichotomized for the binary logistic regression (0 = never or 1 = more than once; for details, see Appendix B).

### 2.3. Statistical Analyses

SPSS for MS Windows Release 22.0 was used to analyse the data, with the maximum significance level set at 0.05. The analysis started with an examination of the descriptive statistics. The primary focus of the analysis was to apply simple bivariate logistic regressions, which helped detect whether these social difference indicators would elevate the risk of a health behaviour. The results are presented as a series of odds. The baseline odds are set to 1.0. An odds ratio > 1.0 indicates that there is a positive association between the factors of interest (serving as a risk factor) and a value < 1.0 indicates the inverse. Confidence intervals (95%) were also calculated for statistically significant relationships based on the criterion that the CIs did not include 1.0.

## 3. Results

The results of the bivariate logistic regression are presented in Table 2 for unhealthy habits. An odds ratio indicates how much higher or lower the risk is in the case of each social difference variable. Gender was significant, with males having nearly twice the odds of reporting not having enough food at home compared to females. In terms of both maternal and paternal education, students whose parents had a primary school education or less or secondary school education had higher odds of reporting not having enough food every month compared to students whose parents had post-secondary educational levels. SES self-evaluation, measured in three categories, found that students who consider themselves lower-middle class or middle class had significantly higher odds of reporting not having enough food every month compared to students in the reference group—upper-middle class or more. Unsurprisingly, students whose parents had lower educational levels (primary school or less) who subjectively assessed themselves as having lower levels of affluence (lower-middle class or lower) were less likely to report having breakfast every day compared to their counterparts.

The bivariate regression results for healthy diet variables are reported in Table 3. Gender was a significant factor in the likelihood of eating fruit daily and vegetables daily. In both cases, males were less likely to report eating healthily compared to females. Students whose parents had primary schooling or less were less likely to eat fruit daily. In the case of paternal and maternal secondary education level, neither was statistically different when compared to the reference category of post-secondary education. In the case of family affluence, students who identified as lower middle class or lower or middle class were less likely to report eating fruit daily compared to their more affluent counterparts. The only significant difference in terms of students eating vegetables daily was in the case of maternal education being secondary school level, which significantly lowered the odds of eating vegetables daily compared to students whose mother had a post-secondary education.

The bivariate regression results for unhealthy eating variables are presented in Table 4. Males were more likely to drink carbonated soft drinks and eat fast food. A paternal education level of primary school or less was significantly associated with students drinking carbonated soft drinks daily. Less affluent students reported lower odds of drinking soft drinks daily. Additionally, paternal education levels of primary school or less or secondary school were associated with being less likely to eat fast food at a restaurant weekly. Both parental education and SES self-evaluation were significant factors in determining odds for these students. Students whose mother had an educational level of primary school or less or secondary school were less likely to eat fast food at a restaurant weekly. Similar to high family SES and its relationship with drinking carbonated soft drinks, students self-reporting their social class as lower middle class or less reported eating less fast food compared to students who were upper-middle or upper class.

The bivariate regression results for hygienic behaviours are presented in Table 5. Male students were significantly less likely to clean or brush their teeth and wash their hands daily compared to female students. The levels of cleaning/brushing teeth daily and hand washing before eating daily were significantly different between students who had paternal and maternal education levels of primary school or less and students whose mother or father had a post-secondary education. In terms of family affluence, those self-reporting as middle class were not significantly different in terms of hygienic behaviour (cleaning or brushing teeth and hand washing daily, *p* > 0.05) compared with those self-reporting as upper-middle class or higher. However, being lower middle class or lower was a significant factor in hygienic behaviour (cleaning or brushing teeth and hand washing daily) compared to students who self-reported being upper middle class or higher.

Table 6 presents the bivariate regression results for smoking and physical activity. Being male was a significant factor in terms of both smoking cigarettes and physical activity. Paternal education was not statistically significant in terms of either smoking or physical activity. Students whose maternal education level was primary school or less were significantly more likely to smoke cigarettes. Finally, those with a self-evaluation of being middle class were a significantly more likely to smoke cigarettes compared to students who self-reported being upper middle class. In terms of physical activity, the levels of those who were lower middle class or less and middle class were both significantly different compared to those who were upper-middle class or higher.

## 4. Discussion

The purpose of this paper was to examine varying health behavioural differences across proxies for social variables among a sample of Jordanian adolescents. Both Arabic and international studies note significant social differences in adolescents’ health behaviours [7,9,10,19,27]. Clearly, more knowledge is needed to better understand these relationships, especially in Arabic countries where only a few studies have investigated multiple health behaviours [19]. Our findings provide preliminary insights into the role of gender, parental education, and family SES in a number of health behaviours among Jordanian adolescents.

The results provide some evidence regarding gender differences in health behaviours. For example, male students reported they did not have enough food at home more frequently than female students. Likewise, males reported eating fewer fruit and vegetables daily compared to their female counterparts; this finding is consistent with previous Jordanian studies [30,33]. In terms of an unhealthy diet, male students were more likely to report drinking carbonated soft drinks and eating more fast food than females. This is also consistent with previous Jordanian studies. Malak [33] reported that males (46.1%) were significantly more likely to drink soft drinks than females (31.7%) and to eat fast food two to seven times daily (33.1% for males; 26.1% for females). Our results are consistent with another international study, although a Jordanian study did not support this finding [13,19].

Male also showed higher levels of unhealthy behaviour when examining specific hygienic behaviours. Male students were significantly less likely to clean or brush their teeth and wash their hands daily when compared with females. This result is consistent with previous research that reported females’ better hygienic behaviour compared to males [19]. Finnish researchers also found that girls brushed their teeth twice daily significantly more often than boys [20]. Finally, male students reported smoking cigarettes and physical activity more frequently than females. These results are consistent with those of previous studies [16,25,26,29].

A possible explanation for these gender differences could certainly be girls’ greater concern with their external appearance and beauty compared to boys, which gives them more motivation to engage in healthy behaviours to maintain their current appearance [19,22].

Our results support the notion that parents’ education level plays an important role in dietary habits. Adolescents whose parents had lower educational levels reported more frequently that they did not have enough food at home or had to skip eating breakfast and reported a lower likelihood of eating fruit and vegetables daily. This is consistent with the results of other studies [9]. In terms of unhealthy eating habits, adolescents whose parents had lower educational levels were less likely to drink carbonated soft drinks and eat fast food, which is consistent with the results of a recent study [40]. This finding might be explained by certain parental attitudes. Parents with high education levels may have a more time-consuming job and spend more time away from home, which may lead to adolescents being able to eat and drink without any parental supervision [19]. Some studies have shown the opposite [12]. More research is needed to clarify the reasons for this finding.

Adolescents whose parents had higher educational levels were more likely to engage in hygienic behaviours than their counterparts. AlBashtawy [21] also reported that Jordanian adolescents whose mother had a higher education level had increased levels of hand washing. Similarly, Virtanen et al. [20] found that adolescents whose parents had a higher level of education brushed their teeth more often than those whose parents had a lower level of education.

In terms of smoking, adolescents whose mother had a higher educational level were less likely to report smoking cigarettes than their counterparts, which is consistent with European data [27] and another study from Saudi Arabia in which adolescents with a highly educated father and mother showed a decreased risk of tobacco use [28]. Further studies should concentrate on these particular social inequalities and their relationship to adolescents’ smoking, particularly in this region.

Adolescents from lower social classes reported not having enough food at home and skipping breakfast more often. In addition, they were less likely to eat fruit and vegetables daily compared to adolescents with higher family affluence. This is consistent with the findings of other studies [10,12]. On the other hand, adolescents with lower family affluence were less likely to drink carbonated soft drinks and eat fast food at restaurants than adolescents with higher family affluence. This is consistent with another study that reported that families with high affluence were characterized by unhealthy eating habits more often than all other groups [36]. This finding is also consistent with another Arabic study [12].

Furthermore, adolescents with higher family affluence were more engaged in hygienic behaviours than their counterparts. This is consistent with another Jordanian study [22], as well as with international studies [23].

Students from higher social classes were more engaged in smoking behaviour than their counterparts from the middle class, which is consistent with the findings of another study [18]. Adolescents with higher family affluence were also more likely to participate in physical activities. This is consistent with a previous Jordanian study and other studies [16,18,29]. These different types of health behaviours may have a common explanation: adolescents with more spending money can more easily afford to buy tobacco and visit sports facilities [41]. This may also mean that they have more access to healthy foods, such as fruits and vegetables. For similar reasons, these adolescents also have more opportunities to smoke or consume more fast food or carbonated soft drinks. 

Our study had some limitations that should be addressed. First, our study was cross-sectional, meaning that it cannot provide any cause-and-effect relationships. Second, the self-reporting bias should be considered when determining levels of subjectivity among the participants. For example, there may be differences in preferences between males and females in food choice or satisfaction with the quality/quantity of food. Third, our study includes adolescents living in one region of Jordan; future research should cover different geographical regions and other countries in the Middle East to confirm our findings. Fourth, we had some difficulties controlling student behaviour in the classroom, especially when it came to girls who were worried about answering sensitive questions about topics such as smoking. However, this study will hopefully encourage the development of additional studies on how social differences can play a role in the determination of Jordanian adolescents’ health behaviours. Finally, although parents’ (both fathers’ and mothers’) job characteristics are much more difficult to include than other SES indicators, future research should try to gain a clearer picture regarding these variables.

## 5. Conclusions

In conclusion, our findings suggest that certain social differences are associated with Jordanian adolescents’ health behaviours. Adolescents with lower parental education or higher perceived SES had a higher likelihood of (1) eating fruit and vegetables; (2) washing their hands and teeth regularly; (3) having adequate food at home and eating breakfast; (4) engaging in physical activities, as well as (5) smoking or engaging in unhealthy eating habits (eating fast food and/or drinking carbonated soft drinks). 

Besides theoretical and scientific considerations for researchers in Jordan and other Arab countries (e.g., for comparison purposes), these findings provide important preliminary evidence for health policy makers and researchers in Jordan that can be useful in the establishment of prevention and intervention health-related programs aimed at reducing unhealthy behaviours among Jordanian adolescents. To our knowledge, there is no program specifically designed for this purpose. In order to encourage healthy schools, the Jordanian Ministry of Education, through education and prevention measures, can implement an active school health program that will go a long way towards the improvement and maintenance of healthy outcomes among Jordanian adolescents.

## Figures and Tables

**Table 1:** Descriptive statistics for study variables.

	n	%
Gender		
Male	1366	49.8
Female	1375	50.2
Paternal Education		
Primary school or less	686	25.0
Secondary school	1283	46.8
Graduate/postgraduate	772	28.2
Maternal Education		
Primary school or less	608	22.2
Secondary school	1326	48.4
Graduate/postgraduate	807	29.4
Family affluence		
Lower-middle or lower	596	21.7
Middle	1383	50.5
Upper-middle or higher	762	27.8
Not having enough food every month		
Yes	1122	40.9
No	1619	59.1
Eating breakfast every day		
Yes	2482	90.6
No	259	9.4
Eating fruit daily		
Yes	2546	92.9
No	195	7.1
Eating vegetables daily		
Yes	2586	94.3
No	155	5.7
Drinking carbonated soft drink daily		
Yes	2365	86.3
No	376	13.7
Eating fast food at a restaurant weekly		
Yes	1888	68.9
No	853	31.1
Cleaning/brushing teeth daily		
Yes	2461	89.8
No	280	10.2
Hand washing before eating daily		
Yes	2672	97.5
No	69	2.5
Smoking cigarettes monthly		
Yes	573	20.9
No	2168	79.1
Physical activity weekly		
Yes	1378	68.3
No	868	31.7

**Table 2:** Sociodemographic factors and dietary habits.

		Not Having Enough Food Every Month			Eating Breakfast Every Day		
Model Variables			OR (95% CI) [n]	Wald	B	OR (95% CI) [n]	Wald
Gender	Male	0.639	1.894 (1.62–2.21) [458]	65.726 ***	0.050	1.051 (0.81–1.35) [1234]	0.146
	Female	1	1 [664]	1	1	1 [1248]	1
Paternal Education	Primary school or less	0.557	1.745 (1.41–2.15) [336]	26.965 ***	−0.393	0.675 (0.48–0.95) [605]	4.971 *
	Secondary school	0.188	1.207 (1.01–1.45) [512]	3.972 *	−0.076	0.927 (0.67–1.28) [1169]	0.216
	Graduate/postgraduate	1	1 [274]	1	1	1 [708]	1
Maternal Education	Primary school or less	0.479	1.615 (1.30–2.00) [289]	19.188 ***	−0.448	0.639 (0.45–0.92) [539]	5.895 *
	Secondary school	0.212	1.236 (1.03–1.48) [543]	5.294 *	−0.276	0.759 (0.55–1.04) [1197]	2.896
	Graduate/postgraduate	1	1 [290]	1	1	1 [746]	1
Family Affluence	Lower-middle or lower	1.214	3.366 (2.69–4.22) [346]	111.235 ***	−0.697	0.498 (0.36–0.70) [502]	16.501 ***
	Middle	0.486	1.626 (1.35–1.97) [554]	25.197 ***	0.179	1.196 (0.86–1.66) [1283]	1.166
	Upper-middle or higher	1	1 [222]	1	1	1 [697]	1

* *p* < 0.05; *** *p* < 0.001.

**Table 3:** Sociodemographic factors and healthy eating habits.

		Eating Fruit Daily			Eating Vegetables Daily		
Model Variables		B	OR (95% CI) [n]	Wald	B	OR (95% CI) [n]	Wald
Gender	Male	−0.631	0.532 (0.39–0.72) [1241]	16.684 ***	−0.730	0.484 (0.34–0.68) [1263]	17.475 **
	Female	1	1 [1305]	1	1	1 [1323]	1
Paternal Education	Primary school or less	−1.026	0.359 (0.24–0.54) [608]	23.261 ***	−0.408	0.665 (0.43–1.04) [639]	3.202
	Secondary school	−0.406	0.666 (0.44–1.00) [1200]	3.783	−0.195	0.823 (0.55–1.24) [1211]	0.869
	Graduate/postgraduate	1	1 [738]	1	1	1 [736]	1
Maternal Education	Primary school or less	−1.103	0.332 (0.22–0.50) [533]	27.479 ***	−0.593	0.552 (0.34–0.89) [567]	5.898 *
	Secondary school	−0.371	0.690 (0.46–1.03) [1242]	3.300	−0.514	0.598 (0.39–0.91) [1243]	5.688 *
	Graduate/postgraduate	1	1 [771]	1	1	1 [776]	1
Family Affluence	Lower-middle class or lower	−2.424	0.089 (0.05–0.15) [486]	74.250 ***	−0.404	0.668 (0.44–1.02) [547]	3.482
	Middle	−0.976	0.377 (0.21–0.66) [1313]	11.480 ***	0.226	1.253 (0.84–0.87) [1320]	1.233
	Upper-middle or higher	1	1 [747]	1	1	1 [719]	1

* *p* < 0.05; ** *p* < 0.01; *** *p* < 0.001.

**Table 4:** Sociodemographic factors and unhealthy eating habits.

		Drinking Carbonated Soft Drinks Daily			Eating Fast Food at a Restaurant Weekly		
Model Variables		B	OR (95% CI) [n]	Wald	B	OR (95% CI) [n]	Wald
Gender	Male	0.264	1.303 (1.12–1.52) [1192]	11.108 **	0.401	1.493 (1.23–1.74) [978]	26.628 ***
	Female	1	1 [1173]	1	1	1 [910]	1
Paternal Education	Primary school or less	−0.360	0.697 (0.52–0.94) [578]	5.483 *	−0.549	0.577 (0.46–0.72) [436]	22.818 ***
	Secondary school	−0.219	0.804 (0.61–1.05) [1104]	2.489	−0.353	0.702 (0.57–0.86) [872]	11.876 ***
	Graduate/postgraduate	1	1 [683]	1	1	1 [580]	1
Maternal Education	Primary school or less	−0.181	0.834 (0.62–1.12) [513]	1.416	−0.389	0.678 (0.54–0.85) [395]	11.166 ***
	Secondary school	0.029	1.030 (0.80–1.33) [1153]	0.050	−0.252	0.778 (0.64–0.94) [902]	6470 *
	Graduate/postgraduate	1	1 [699]	1	1	1 [591]	1
Family Affluence	Lower-middle or lower	−0.698	0.497 (0.37–0.68) [481]	19.824 ***	−1.573	0.207 (0.16–0.27) [302]	153.696 ***
	Middle	−0.229	0.795 (0.60–1.05) [1203]	2.607	−0.808	0.446 (0.36–0.56) [952]	51.108 ***
	Upper-middle or higher	1	1 [681]	1	1	1 [634]	1

* *p* < 0.05; ** *p* < 0.01; *** *p* < 0.001.

**Table 5:** Sociodemographic factors and hygienic behaviours.

		Cleaning or Brushing Teeth Daily			Hand Washing before Eating Daily		
Model Variables		B	OR (95% CI) [n]	Wald	B	OR (95% CI) [n]	Wald
Gender	Male	−1.209	0.299 (0.26–0.35) [1130]	225.479 ***	−0.651	0.521 (0.44–0.62) [1311]	57.920 ***
	Female	1	1 [1331]	1	1	1 [1361]	1
Paternal Education	Primary school or less	−0.659	0.517 (0.37–0.72) [581]	15.622 ***	0.455	1.576 (0.85–2.94) [670]	2.047
	Secondary school	0.007	1.007 (0.73–1.39) [1174]	0.002	0.639	1.894 (1.10–3.27) [1258]	5.238 **
	Graduate/postgraduate	1	1 [706]	1	1	1 [744]	1
Maternal Education	Primary school or less	−0.418	0.658 (0.48–0.91) [523]	6.271 **	−0.617	0.540 (0.27–0.99) [582]	4.042 *
	Secondary school	0.100	1.106 (0.82–1.49) [1209]	0.428	0.269	1.308 (0.71–2.40) [1302]	0.749
	Graduate/postgraduate	1	1 [729]	1	1	1 [788]	1
Family Affluence	Lower middle or lower	−1.215	0.297 (0.21–0.42) [480]	46.515 ***	−0.749	0.473 (0.26–0.86) [567]	6.016 **
	Middle	−0.215	0.806 (0.57–1.14) [1270]	1.515	0.403	1.479 (0.80–2.81) [1361]	1.577
	Upper middle or higher	1	1 [711]	1	1	1 [744]	1

* *p* < 0.05; ** *p* < 0.01; *** *p* < 0.001.

**Table 6:** Sociodemographic factors and health behaviours.

		Smoking Cigarettes Monthly			Physical Activity Weekly		
Model Variables		B	OR (95% CI) [n]	Wald	B	OR (95% CI) [n]	Wald
Gender	Male	1.309	3.704 (3.02–4.55) [424]	156.549 ***	0.206	1.229 (1.05–1.44) [964]	2.297 ***
	Female	1	1 [149]	1	1	1 [909]	1
Paternal Education	Primary school or less	0.216	1.241 (0.97–1.59) [161]	2.859	−0.075	0.928 (0.75–1.16) [457]	0.449
	Secondary school	0.023	1.023 (0.82–1.28) [259]	0.041	0.048	1.049 (0.87–1.27) [889]	0.237
	Graduate/postgraduate	1	1 [153]	1	1	1 [527]	1
Maternal Education	Primary school or less	0.308	1.361 (1.06–1.75) [154]	5.774 **	−0.118	0.889 (0.71–1.11) [404]	1.054
	Secondary school	−0.031	0.969 (0.78–1.21) [258]	0.077	−0.011	0.989 (0.82–1.20) [912]	0.014
	Graduate/postgraduate	1	1 [161]	1	1	1 [557]	1
Family Affluence	Lower middle or lower	0.244	1.277 (0.96–1.64) [159]	3.687	−0.559	0.551 (0.44–0.70) [369]	25.219 ***
	Middle	−0.280	0.755 (0.61–0.94) [245]	6.261 **	−0.345	0.708 (0.58–0.86) [935]	11.652 ***
	Upper middle or higher	1	1 [169]	1	1	1 [569]	1

** *p* < 0.01; *** *p* < 0.001.

## Data Availability

The data presented in this study are available on request from the corresponding author.

## Appendix A. The Multi-Stage Technique Used in the Study

At the first stage, after using a simple random sampling technique in the form of a lottery technique, a sample of four districts (Bani Obaid, Al-Ramtha, Irbid Qasabt, and Al-kora district) was selected from a list of eight districts in Irbid governorate. Following this, we obtained the names and number of public and private schools in these districts. In total, 156 secondary schools were identified. The schools were stratified by gender (64 female schools and 71 male schools) and type of school (21 private and 135 public). From the 135 public schools, 16 schools were randomly selected (8 male schools and 8 female schools). Four schools (2 female schools and 2 male schools) were randomly selected from every district using a stratified sampling technique. From the 21 private schools, 6 private schools were randomly selected. From each of the selected public schools (n = 16 schools), a sample of five classes (8th, 9th, 10th, 11th, and 12th grade) was selected using a simple random sample technique. The total number of students was 135 from every school. All students in the randomly selected classes were asked to participate in the study. The final sample size from public schools was n = 2141 students. From each of the selected private schools (n = 6 schools), a sample of five classes (8th, 9th, 10th, 11th, and 12th class) was selected using a simple random sample technique. These schools contain male and female students. The total number of students was 100 from every school. All students in the randomly selected class were asked to participate in the study. The final sample size from private schools was n = 600 students.

## Appendix B. Measures of Health Behaviour in the Study

To assess dietary behaviour, the questions included the following: (1) ‘During the past 30 days, how often did you go hungry because there was not enough food in your home?’ where the responses were coded as 0 (never) or 1 (yes: rarely, sometimes, most of the time, or always); (2) ‘During the past 30 days, how often did you eat breakfast?’ where the responses were coded as 0 (never) or 1 (yes: rarely, sometimes, most of the time, or always); (3) ‘During the past 30 days, how many times per day did you usually eat fruit, such as apples, bananas, or citrus fruits?’ where the responses were coded as 0 (never) or 1 (yes: eating fruits ≥ 1 times/day); (4) ‘During the past 30 days, how many times per day did you usually eat vegetables, such as tomato, cucumber, spinach, or eggplant?’ where the responses were coded as 0 (never) or 1 (yes: eating vegetables ≥ 1 times/day); (5) ‘During the past 30 days, how many times per day did you usually drink carbonated soft drinks, such as Coke, Pepsi, Coca Cola, 7-Up, or Fanta?’ where the responses were coded as 0 (never) or 1 (yes: drinking ≥ 1 times/day); (6) ‘During the past 7 days, on how many days did you eat at a fast food restaurant, such as McDonald’s, Boston Fried Chicken, or Burger King?’ where the responses were coded as 0 (never) or 1 (yes: eating ≥ 1 d/w).

For hygienic behaviour, the questions included the following: (1) ‘During the past 30 days, how many times per day did you usually clean or brush your teeth?’ where the responses were coded as 0 (never) or 1 (yes: cleaning or brushing teeth ≥ 1 times/d); (2) ‘During the past 30 days, how often did you wash your hands before eating?’ where the responses were coded as 0 (never) or 1 (yes: rarely, sometimes, most of the time, or always).

For tobacco use, the question was: ‘During the past 30 days, on how many days did you smoke cigarettes?’ where the responses were coded as 0 (no) or 1 (yes it happened, which means smoking cigarettes ≥ 1 or 2 days/m). For physical activity, the question was: ‘During the past 7 days, on how many days were you physically active for a total of at least 60 min per day?’ where the responses were coded as 0 (no) or 1 (yes it happened, which means physically active ≥ 1 day/w).
